# Filing Teeth—Dr. Arthur's Method

**Published:** 1869-02

**Authors:** James Truman


### SELECTED ARTICLES.

---

### ARTICLE V.

### *Filing Teeth—Dr. Arthur's Method.*

By JAMES TRUMAN, D. D. S.

The discussion of this subject, at the present time, in the various Dental Associations, is but another evidence of the pisposition to adopt at one period practices that have been discarded at a previous as valueless or absolutely injurious. It is undoubtedly true, that in all the modes of practice that have at various times have been introduced in our profession, and been abandoned, there has been a large admixture of truth with a still greater amount of error. That this is true of filing teeth, must be evident to any one who will take the trouble to read the history of dentistry for the past hundred years. It is within the knowledge of all, that filing was mainly practiced as a preventive of caries during the last, and the first twenty years of the present century. The skillful use of this instrument entitled the individual to rank high as an operator with the public, in France, England, and in this country. But we find writers in the latter part of the last century condemning the practice as dangerous and destructive to the teeth. Berdmore,* writing in 1770, speaks thus of this operation: "It cannot be supposed that any man is so lost to shame and humanity as to expose his patient to pain and inconvenience during life, merely for the sake of a trifling fee. The indiscriminate filing of teeth, so common at present, should be imputed only to ignorance, and may, I hope, be checked, by placing the subject in a clear light, and by *drawing the line* to distinguish where it may and where it may not be practiced with safety." He then proceeds to give his views when it is expedient to use this instrument, which generally accord with present practices.

This able author had the clearest and best practical ideas of his day, and in many respects we have failed to improve upon the modes adopted and promulgated in his works. His opinion is, therefore, to be received with weight in considering the effects of this practice. But, while it is evident that the per centage of failures at that and subsequent periods must have been largely in excess of successes, we, it seems to me, have no reason therefore to conclude that no permanent good resulted from the practice. A large allowance must be made for the modes adopted, and for the evident want of knowl-

---

* Treatise on the Disorders and Deformities of Teeth and Gums, by Thomas Berdmore, London, 1770.

edge in practical details and of the minute structure of the teeth.

Gradually, separating teeth by the file fell into disuse, until many excellent operators almost altogether abandoned it. I have long been satisfied that this is a practical error—believing that, where judiciously used, it is one of our most valuable instrumeuts. It must be evident to every practitioner that a wholesale destruction of teeth is going on yearly, from a want of knowledge when and how teeth should be separated by the file. The prejudices that surround this subject prevent a clear judgment, and lead to fallacious reasoning.

The propositions announced by Dr. Arthur, as a basis of reasoning and practice, have already been published in this Journal.—Eds.

The position laid down by Dr. A., that when caries attacks the superior incisors previously to the twelfth year, that it will also attack the proximate surfaces of all the teeth, except the inferior incisors, cannot be successfully controversed. The teeth, in their various degrees of development, are necessarily subjected to the same influences that operate either to the benefit or injury of the structure. If, therefore, caries be found on the proximate surfaces of incisors, it may reasonably be inferred, that sooner or later it will make its appearance on all the teeth mentioned. Where this result does not follow, it must be from one or two causes:

1st. That the proximate surfaces of the teeth are not closely in contact, and admit freely the passage of the brush or fluids between.

2d. That the patient, by the exercise of constant care, has kept the surfaces free from all collections.

When decay attacks the incisors at a later period in life, it does not necessarily follow that the surfaces mentioned will be affected.

If, then, this position be true, it becomes a question of serious import whether, if one of the incisors be decayed at this early period, we should at once proceed to make a separation in the balance to avoid the results almost certain to follow? I understand Dr. A. to answer, "that it is the best practice to make the separation before the progress of the caries has rendered this method of treatment impossible."

While I endorse the fact that caries will attack these anterior teeth, I do not think it advisable to enter at once upon the separation of all the proximate surfaces anterior to the bicupids. There is always a doubt whether this result will follow, and we should give the teeth the benefit of this doubt, and wait until decay manifests itself. I make exception in the case of these anterior teeth, because they, above all others, are immediately under the supervision of the operator and patient; very few of the latter will permit caries to make any progress before calling the attention of the dentist to the fact. Separation can then be made of the proper form, and the simple cavity filled, producing no disfigurement to the tooth.

The same reasoning and mode of practice does not, it seems to me, hold good with the bicuspids. Further removed from observation, and closely pressed together on their proximate surfaces, the ordinary opportunities for observation are not present.

Without entering into the theories of caries, that have at various times been promulgated, I may say in brief, that the destructive agent, having once effected a lodgment, soon breaks down the tubular structure of the dentine, and that is removed with great rapidity, without a corresponding loss of the hard enamel tissue. This is the process common to all the teeth.

We find in the proximate surfaces of bicuspids, superior and inferior, caries penetrating the teeth at the point of juncture of the surfaces, or slightly above it. It will then pass into the dentine, and very commonly destroy a large interior surface, before either dentist or patient is aware of the fact. To the properly educated eye, this progress of caries is manifested from its first entrance in the dentine, by the slight change of color of the enamel. If this infallible sign were always observed and attended to, there would be but little difficulty in the management of these teeth, but, that it is almost entirely neglected, is patent to all observers. The teeth are allowed to remain until the cavity is exposed by the breaking of the surrounding wall, or the pulp is nearly or entirely exposed, producing pain.

Caries may, however, be present in the enamel and give no indication; indeed, I think it may truthfully be asserted that the majority of patients have these teeth at some stage of disease.

Now, admitting these to be facts, what would seem to be the proper course? Certainly the whole duty of the operator has not been performed if he neglects to separate these teeth thoroughly before leaving the case. This has been my practice for a long time, and one forced upon me by the observations of experience. Hence, whether the blue tinge be present or not, the teeth are filed freely, fully believing that, if there be no decay, the separation made will go very far to prevent it, and, if it be present, I am equally prepared to to meet it.

The objection to filing the bicuspids, by those who admit their constant liability to decay, is based on the fact that it involves the destruction of so much good tissue, and that this cannot be done without injuring the shape of the tooth at the masticating surface, the mode usually adopted being to remove mainly from the lingual and palatine surfaces, and but little from the buccal. I do not see the force of the objection. That there will be a trifling disfigurement is admitted, but it is almost entirely hidden from view. The advantages derived more than counterbalances this objection.

The other and more important one is, that all the filed surfaces are more liable to attack of caries than those covered by enamel. This would, perhaps, be true in practice, as it seems reasonable in theory, were it not that the fact is well known that an abraded surface of dentine never remains in the condition of a tissue with a series of open-mouthed tubes.

We see this beautifully illustrated in the deposition of secondary tissue in the pulp, as fast as attrition removes the crown in its near appproach to that organ. Here the constant but slight irritation renews the formative process, and a further deposition of calcareous particles and the formation of the irregular tissue, called osteo or secondary dentine, takes place. This aproximates dentine in its formation, but has none of its regular tubular structure.

Another illustration may be found in the increased deposition of cementum in exostosis, produced by constant irritation. A better illustration may be seen in those masticating surfaces extensively worn by opposing teeth. The surface here presents almost the density and polish of the enamel. The same result is witnessed in the arrestation of caries by the consolidation of the tubes, with their contents, into one solid mass.

Reasoning, therefore, from analogy, all filed surfaces must, to a greater or less extent, partake of a similar character. Consolidation must necessarily take place, opposing any further encroachments of disease, with ordinary care.

If this be admitted, what possible injury can result in the use of the file, if the surface be subsequently properly polished ? I have no hesitation in asserting that no evil results can follow.

Having considered some of the objections, what may we hope for in the way of benefits ? In the first place, we obviate the danger of excessive loss of structure, which delay invariably occasions, in these important teeth. We give the patient ample opportunity to free the teeth from all particles of food, secretions, &c. In a word, we insure the teeth for an indefinite future of usefulness. On the other hand, he who neglects them until decay has manifested itself, and some portion of the walls have broken away, has a tooth always unreliable, and one infinitely more of a disfigurement to the mouth.

The rule must be observed to form the space of a shape that will not only prevent the proximate surfaces from coming together, but that they may be readily freed from all secretions. To save the appearance of the tooth, the broadest separation must necessarily be made toward the palatine and lingual walls.

The whole process of filing may, and in all probablility will, prove a failure, if the finishing process be not performed thoroughly. Any roughness left furnishes a lodgment for the materials necessary to produce the commencement of caries. The same care should be exercised here as in the finishing of fillings, and for the same reason. The most active ingredients in the oral secretions are those microscopic in their character; hence, depressions, however minute they may be, will probably cause a renewal of disease.—*Dental Times.*

---

